# Case of Nasal Enlargement Successfully Treated

**Published:** 1843-03

**Authors:** Chas. Clay


*Case of Nasal Enlargement successfully treated.*—By Dr. Chas. Clay.—This was a case of peculiar enlargement of the nose, in a young lady, unaccompanied with pain or any other inconvenience than the size. Besides constitutional treatment, Dr. Clay made pressure on the organ, by means of a mould made of plaster of Paris, which was useful not only by pressing uniformly, but also by its mere weight. The mould was secured to the head by different tapes, which were applied so as to increase the pressure. In a week the mould was found too large, and a second one was made; a third, fourth, and fifth were obliged to be made, as the nose diminished in size, till it regained its natural dimensions.

*London Lancet.*

